# Effect of an intervention to promote contraceptive uptake on incident pregnancy: a randomized controlled trial among HIV positive couples in Zambia

**DOI:** 10.1186/1742-4690-9-S2-P211

**Published:** 2012-09-13

**Authors:** K Wall, B Vwalika, L Haddad, N Htee Khu, C Vwalika, W Kilembe, E Chomba, R Stephenson, D Kleinbaum, A Nizam, I Brill, A Tichacek, S Allen

**Affiliations:** 1 Emory University, Atlanta, GA, USA; 2 Rwanda Zambia HIV Research Group, Atlanta, GA, USA; 3 University of Alabama at Birmingham, Birmingham, AL, USA

## Background

Prevention of unintended pregnancy, especially among HIV positive couples and in settings where both HIV and total fertility rates are high, is a critical public health initiative.

## Methods

A factorial randomized controlled trial evaluated the effect on incident pregnancy of two interventions (a “Methods” and “Motivational” intervention) to promote long-term contraceptive use among HIV serodiscordant and concordant positive couples (N = 1060) identified from CVCT clinics in Lusaka, Zambia.

## Results

Couple baseline serostatus and contraception usage were both individual effect measure modifiers (p<0.0001). Among couples in which the woman was not using a contraceptive method at baseline (N=782), there was no significant effect of the interventions overall or when stratifying by couple serostatus on incident pregnancy. Among couples in which the woman was using a contraceptive method at baseline, concordant positive couples (HR = 0.20; 95%CI: 0.08-0.53), and couples in which the woman was HIV positive at baseline (HR = 0.21; 95%CI: 0.09-0.51) who received “Methods + Both” interventions - which combined information on contraceptive methods and motivational messages for future planning behaviors - were at significantly decreased hazard for pregnancy relative to those receiving “Motivational + Control” interventions -- which provided motivational messages for future planning but not information on contraceptive methods.

## Conclusion

An educational intervention promoting long-term contraceptive method uptake among HIV positive couples is successful at decreasing time to pregnancy in the context of couples’ HIV testing, particularly among women who are HIV+ and already using a contraceptive method. A combination of motivational messages for future planning behaviors and information on long-term contraceptive methods appears to be the best intervention for reducing incident pregnancy among concordant positive and serodiscordant couples. Further work is needed to understand the interventions appropriate for women who are currently not contraceptive users.

